# The role of primary cilia in mouse adrenal and zebrafish interrenal development

**DOI:** 10.1186/2046-2530-1-S1-P64

**Published:** 2012-11-16

**Authors:** K Cogger, L Guasti, R Ashworth, C Brennan, P Beales, V Marion, P King

**Affiliations:** 1 Queen Mary University of London, UK; 2 University College London, UK; 3 Université de Strasbourg, France

## 

Primary cilia play key roles in development, cell signalling and cancer, and are involved in signal transduction pathways such as Hh and Wnt signalling. The adrenal cortex produces steroid hormones essential for controlling homeostasis and mediating the stress response. Signalling pathways involved in the process of its development and differentiation are still being identified but include Hh and Wnt, and adrenal development is thus likely to require cilia. I have demonstrated that inhibiting cilia formation, using siRNA targeted to different ciliary components, results in reduced differentiation of the human adrenal carcinoma cell line H295R towards a zona glomerulosa (zG)-like phenotype. These data suggest that primary cilia play a key role in adrenal differentiation, but which signalling pathways are involved still remains unclear. I have also discovered that adrenals from Bardet-Biedl syndrome (BBS) mice, the most prominently studied ciliopathy, have thin capsules, the proposed adrenal stem cell niche, and abnormal histology, while zebrafish embryos injected with morpholinos targeting BBS genes show delayed and reduced expression of ff1b, a marker of interrenal tissue. These data further suggest a role for primary cilia in adrenal development and maintenance. These studies are the foundation for elucidating the role of primary cilia in the development and function of the adrenal gland, and furthering our understanding of adrenocortical development. This promises to lead to improved management of adrenal dysfunction, and demonstrating that adrenal defects are a characteristic of ciliopathies will potentially inform new strategies for patient care.

